# Valorization of Mushroom Residues for Functional Food Packaging

**DOI:** 10.3390/ijms262210870

**Published:** 2025-11-09

**Authors:** Gréta Törős, Hassan El-Ramady, Neama Abdalla, Tamer Elsakhawy, József Prokisch

**Affiliations:** 1 Institute of Animal Science, Biotechnology and Nature Conservation, Faculty of Agricultural and Food Sciences and Environmental Management, University of Debrecen, Böszörményi Street 138, 4032 Debrecen, Hungary; toros.greta@agr.unideb.hu; 2 Doctoral School of Animal Husbandry, Faculty of Agricultural and Food Sciences and Environmental Management, University of Debrecen, Böszörményi Street 138, 4032 Debrecen, Hungary; 3 Soil and Water Department, Faculty of Agriculture, Kafrelsheikh University, Kafr El-Sheikh 33516, Egypt; hassan.elramady@agr.kfs.edu.eg; 4 Plant Biotechnology Department, Biotechnology Research Institute, National Research Centre, 33 El Buhouth St., Dokki, Giza 12622, Egypt; na.abdel-aal@nrc.sci.eg; 5 Microbiology Department, Soil, Water and Environment Research Institute, Sakha Agricultural Research Station, Agriculture Research Center, Kafr El-Sheikh 33717, Egypt; drelsakhawy@arc.sci.eg

**Keywords:** mushroom residues, bioactive compounds, sustainable packaging, circular economy, green extraction

## Abstract

The mushroom industry generates a substantial amount of residues each year, encompassing materials such as processing residues and spent substrates. Much of this biomass is discarded, despite its richness in valuable compounds. Mushroom residues contain bioactive substances including β-glucans, phenolic compounds, proteins, and dietary fiber, all of which are well known for their antioxidant and antimicrobial properties. While fruit and vegetable residues have been extensively explored as raw materials for eco-friendly packaging, mushroom-derived residues remain a largely underutilized resource. Recent studies have highlighted their potential as a renewable source of functional ingredients for sustainable food packaging. By applying green extraction technologies such as ultrasound- or microwave-assisted methods, researchers can recover stable bioactive compounds and incorporate them into biodegradable polymers. Early results are promising: packaging films enriched with mushroom residue extracts demonstrate improved mechanical strength, enhanced barrier properties, and added bioactivity. This strategy aligns with the principles of the Circular Economy, simultaneously reducing environmental impact and adding value to materials that were previously discarded. Although further optimization is needed, particularly regarding extraction efficiency, compound stability, and scalability, the valorization of mushroom residues represents a promising pathway toward the next generation of sustainable, eco-friendly packaging materials.

## 1. Introduction

The escalating global concern over plastic pollution, combined with growing environmental awareness, is prompting the packaging industry to transition toward sustainable, bio-based alternatives [1]. Although petroleum-based plastics continue to dominate the food packaging sector due to their affordability, versatility, and durability [2], their environmental persistence and role in microplastic contamination present serious ecological risks [3]. As regulatory pressure on single-use plastics intensifies and consumers increasingly seek eco-friendly solutions, both researchers and industries are shifting their focus to renewable feedstocks for biodegradable packaging materials [4].

At the same time, the food processing industry produces considerable quantities of underutilized residues, many of which hold untapped potential as raw materials [5]. Traditionally, these residues have been utilized in low-value applications, such as animal feed, compost, or energy recovery [6]. However, the transition toward a circular economy has brought renewed attention to their value, particularly for their rich content of functional compounds with applications in high-performance packaging [7]. By utilizing such residue streams, it becomes possible not only to reduce the environmental burden of disposal but also to develop biopolymeric films and coatings that can extend shelf life and lessen dependence on synthetic additives [8].

Among various agri-food sectors, the mushroom industry stands out due to its rapid global expansion and the significant amount of biomass it discards [9]. Global mushroom production exceeds 9 million tonnes annually, driven by consumer demand for their nutritional and health benefits. However, cultivation and processing result in substantial residues, such as spent mushroom substrate (SMS), degraded sporophores, stipes, and litter, that are often relegated to low-efficiency applications or left unutilized [10,11,12]. With residue generation surpassing 5 million tonnes per year, the industry faces a dual challenge and opportunity in managing and valorizing these materials [13].

Notably, mushroom residues are rich in bioactive compounds, including β-glucans, phenolics, proteins, chitin, and dietary fibers. These components are known for their antioxidant, antimicrobial, and health-promoting effects [12,14], and can enhance the functionality of biodegradable packaging. For example, β-glucans offer immunomodulatory and antioxidant benefits [15], phenolic compounds protect against oxidation, and proteins can improve the structural integrity of polymer films [16]. Together, these features make mushroom residues a promising source for active packaging solutions that can reduce environmental impact while maintaining food quality.

Valorizing mushroom residues supports circular economy goals by turning residue into value-added products, reducing reliance on landfills, and creating economic opportunities [17]. The application of green extraction techniques, such as ultrasound- and microwave-assisted methods, further contributes to sustainability by enabling efficient, low-impact recovery of bioactive compounds [18,19].

This review explores the potential of mushroom industrial residues as sources of functional compounds for food packaging. It highlights their antioxidant and antimicrobial capabilities, assesses their incorporation into biodegradable polymers, and addresses challenges such as compound stability, industrial scalability, and regulatory acceptance.

## 2. Mushroom Industry, and Characterization of Mushroom Residues

The global mushroom industry has experienced robust growth, driven by increasing consumer demand for nutritious, plant-based foods and their applications in functional foods and pharmaceuticals. According to FAOSTAT data, global production of mushrooms and truffles rose from about 0.5 million tons in 1961 to over 10.24 million tons by 2017, with further expansion continuing in recent years [20]. More recently, projections suggest global mushroom production is approaching ~44 million tons. China dominates production, accounting for over 90% of global output. This expansion, however, generates substantial residual streams, necessitating sustainable valorization strategies to mitigate environmental impacts such as greenhouse gas emissions from improper disposal and resource loss [21].

### 2.1. Residue Types

Mushroom production yields diverse residual streams, primarily lignocellulosic residues rich in organic matter, nutrients, and residual mycelium. The predominant residue is spent mushroom substrate (SMS), the nutrient-depleted lignocellulosic material (e.g., composted straw, manure, or sawdust) remaining after multiple harvest flushes, which constitutes the bulk of post-cultivation residues [22]. Other key types include:

- Trimming residues—Discarded stipes (stems), caps, and misshapen or undersized mushrooms, often from quality control during harvesting and packaging, representing 5–10% of fresh yield.
- Extraction residues—Leftover solids from processing mushroom extracts for bioactive compounds (e.g., polysaccharides or beta-glucans), typically fiber-rich and low in soluble nutrients.
- Mycelium residues—Excess fungal biomass from spawn production or failed cultivation batches, containing high protein and enzyme content.

These residues are generated across cultivation phases, with SMS being the most voluminous due to its role as the primary growth medium [22].

### 2.2. Quantitative Estimates (Global and Regional Volumes)

For every 1 kg of fresh mushrooms produced, approximately 4–5 kg of spent mushroom substrate is generated [23]; in certain cases, 5 to 6 kg SMS is observed per kg of mushrooms [24]. Using 2017 baseline global production of 10.24 million tons, this could translate to ~40–60 million tons of SMS generated globally [25]. Regionally, China alone accounts for a significant share of that SMS, in line with its dominance in mushroom production [26]. Trimming residues and extraction residues remain relatively small fractions compared to SMS [24]. These figures underscore the urgency of valorization, as traditional disposal methods (e.g., landfilling or incineration) incur high costs and environmental risks [21].

### 2.3. Physicochemical and Microbiological Characteristics Relevant to Valorization

SMS and related residues exhibit physicochemical profiles conducive to valorization as biofertilizers, animal feeds, bioenergy feedstocks, and bioremediation agents, owing to their lignocellulosic composition and residual nutrients [22,24]. Key characteristics are summarized in Table 1.

Microbiologically, SMS harbors lignocellulolytic bacteria and fungi, supporting degradation, suppressing pathogens, and enabling recovery of bioactive compounds such as polysaccharides [22]. Trimming residues often have higher protein (15–25% for dry matter) and moisture (>80%), making them suitable for feed formulations, while extraction residues are fiber-rich and favor biochar or energy uses [24]. These traits position mushroom residues as assets in a circular economy, reducing disposal burdens while enabling high-value recovery [21,22].

**Figure 1:**
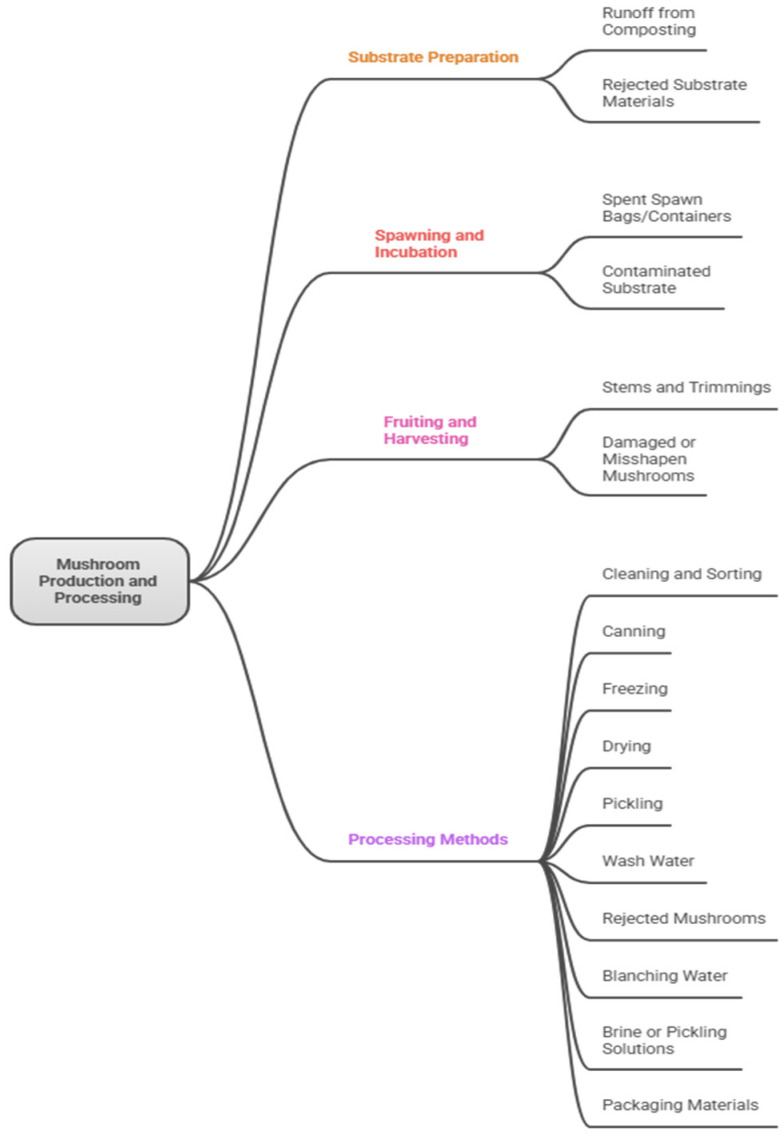
Mushroom production and processing: residues stream and management.

**Figure 2:**
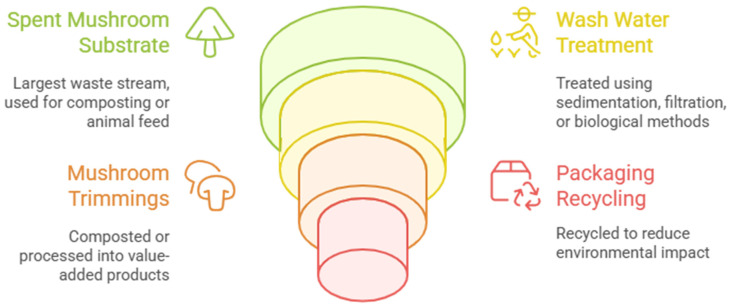
Circular valorization pathways of mushroom industry residues, highlighting the reuse of spent mushroom substrate, mushroom trimmings, wash water, and packaging materials through composting, recycling, and treatment processes.

Figure 1 shows the mushroom production and processing with focus on the mushroom residues stream and management, whereas Figure 2 suggests the pathway of using of SMS in packing recycling.

## 3. Molecular and Functional Constituents of Mushroom Residues

### 3.1. Major Bioactive Compounds (β-Glucans, Phenolics, Proteins, Chitin)

Mushroom residues have garnered growing scientific interest due to their rich molecular diversity and multifaceted functionality, with promising implications for both health-related applications and sustainable material development. These residues are a valuable source of bioactive compounds, including polysaccharides (notably β-glucans), phenolic compounds, proteins, peptides, chitin, chitosan, and minor components such as lipids, sterols, and terpenoids, each contributing unique biochemical and technological properties [27,28].

#### 3.1.1. Polysaccharides

Among these, polysaccharides, particularly β-glucans, have been the subject of extensive research. Typically composed of β-(1→3)-D-glucans with β-(1→6) branches, their molecular weight and structure vary depending on fungal species and extraction methodology. β-glucans are well recognized for their immunomodulatory, antitumor, and genoprotective activities [15,29]. For example, Boulaka et al. (2020) demonstrated that β-glucans derived from edible mushrooms exhibited genoprotective effects following in vitro fermentation by human gut microbiota, reinforcing their prebiotic potential [30]. In a complementary study, Hasan and Abdulhadi (2023) characterized β-glucans extracted from wild *Pleurotus ostreatus*, identifying key structural features and functional derivatives relevant to both biopackaging and health-supportive innovations [31].

Chitin and its deacetylated derivative chitosan, integral components of fungal cell walls, are especially valued for their film-forming ability and antimicrobial effects. The degree of deacetylation (DDA) plays a critical role in determining solubility and biological activity [32]. Mushroom-derived chitosan, often characterized by high DDA, is therefore well-suited for creating biodegradable films with intrinsic pathogen-inhibiting properties [33,34].

#### 3.1.2. Phenolic Compounds

Phenolic compounds, such as phenolic acids and flavonoids, also significantly enhance the antioxidant profile of mushroom residues. These molecules occur in free, conjugated, or bound forms, often requiring targeted fractionation for effective isolation and characterization [35]. For instance, Erbiai et al. (2023) investigated two wild edible mushrooms from Morocco and identified syringic acid as the predominant phenolic, while rutin and quercetin were absent. Their findings highlighted the potent antioxidant and anti-inflammatory properties of these phenolics, underscoring their potential for integration into active packaging systems designed to extend shelf life and enhance food safety [36].

#### 3.1.3. Proteins and Peptides

Proteins and peptides retained in mushroom residues often maintain bioactivity even after processing. These include antioxidant peptides and residual enzymes with hydrolytic capabilities [37]. Supporting this, Hasan and Abdulhadi (2023) elucidated the biochemical complexity of *Pleurotus ostreatus*, detecting protein fragments with both antimicrobial and antioxidative properties, attributes particularly pertinent to the development of next-generation bioactive packaging materials [38].

These peptides can be obtained from agricultural mushroom residues, offering an effective way to add value to what would otherwise be residue. One notable example is copsin, a naturally occurring defensin produced by *Coprinopsis cinerea*. Copsin has attracted attention for its strong antibacterial properties and its remarkable stability even under challenging conditions such as high temperatures or acidic environments. It targets lipid II, a key component of bacterial cell wall synthesis, and has proven particularly effective against Gram-positive bacteria [39,40].

**Figure 3:**
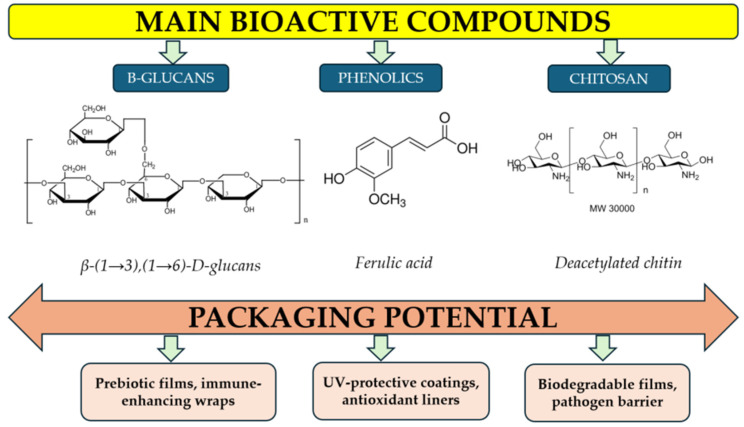
Bioactive Compounds in Mushroom Residues.

Because it originates from fungi, copin may also integrate more naturally with other fungal-based materials, such as chitin or β-glucans [41], potentially enhancing the cohesion and performance of composite biopolymer films [42]. The main bioactive compounds can be extracted from mushroom residue (with their molecular structures) and are responsible for packaging potential, as shown in Figure 3.

### 3.2. Minor Components and Synergistic Effects

Although present in smaller quantities, minor constituents such as lipids, sterols (e.g., ergosterol), and terpenoids also contribute to the overall bioactivity of mushroom residues. These molecules exhibit antioxidant and antimicrobial functions and, as reported in several studies [43], may act synergistically with major compounds to enhance the functional performance of bio-based packaging materials.

Although direct applications remain rare, the known bioactivity of ergosterol and sterol derivatives from mushrooms (e.g., ergosterol peroxide) supports their candidacy as minor synergistic enhancers in composite films [44]. In packaging contexts more broadly, active compounds (including volatile terpenoids) have been shown to improve antioxidant capacity demonstrably, slow microbial growth, and enhance barrier properties when blended with polymer matrices [45]. Field-specific examples include the active packaging of *Agaricus bisporus* with antioxidant extracts, which yields slower browning, improved water retention, and extended shelf life [46]. While empirical evidence is limited, these data suggest promising potential for minor fungal compounds to act synergistically in bio-based packaging systems.

### 3.3. Implications for Packaging Functionality

The molecular and functional diversity of mushroom residues offers a promising foundation for developing novel bio-based materials, particularly in sustainable food packaging. The integration of polysaccharides, phenolics, proteins, and minor bioactives can impart multifunctionality to packaging films, enhancing both food preservation and environmental sustainability [47]. As research progresses, optimizing extraction techniques, enhancing compound stability, and scaling up production will be crucial to realizing the full potential of these bioresources [48,49].

## 4. Functional Properties of Extracts and Derivatives

### 4.1. Overview of Functional Roles in Food Packaging

Transforming mushroom residues into valuable packaging components exemplifies the potential of sustainable material science. Functional packaging today goes beyond passive containment to actively preserve and protect food, extending shelf life and maintaining quality [50]. Bioactive compounds, such as antioxidants, antimicrobials, and barrier enhancers are incorporated into packaging materials to achieve these effects [51].

Mushroom-derived compounds have emerged as potent multifunctional additives. These include natural polymers like chitin and chitosan, phenolic compounds, and polysaccharides, which naturally evolved as protective agents in fungi [52]. These natural polymers and secondary metabolites, evolved over millions of years to protect fungal organisms from environmental stresses, now offer similar protective benefits when incorporated into packaging systems [53]. For example, chitin-chitosan complexes offer structural support and antimicrobial action, while phenolics contribute antioxidant and UV-absorbing properties [54]. β-glucans, another key component, enhance film formation and maintain bioactivity, making them ideal for packaging systems requiring integrated functional performance [55].

Unlike synthetic additives, mushroom-based bioactives can simultaneously provide multiple benefits within a single formulation [56]. Unlike synthetic additives that typically target single functions, mushroom extracts can provide antioxidant, antimicrobial, and barrier properties within the same formulation [57]. This multifunctionality simplifies design and minimizes additive load, aligning with clean-label trends and consumer preferences for natural food packaging [58].

### 4.2. Antioxidant Properties

The antioxidant prowess of mushroom-derived compounds lies in their sophisticated molecular architecture, fine-tuned by evolutionary pressures to combat oxidative stress in natural environments [59]. Mushroom polysaccharides, particularly β-glucans, demonstrate remarkable radical-scavenging activities through multiple mechanisms [60]. Petraglia et al. [61] reported that polysaccharide fractions from *Pleurotus eryngii* exhibited DPPH IC_50_ values of 0.52 ± 0.02 mg·mL^−1^, superoxide IC_50_ of 1.15 ± 0.09 mg·mL^−1^, and hydroxyl radical IC_50_ of 0.89 ± 0.04 mg·mL^−1^, demonstrating potent multi-radical scavenging capacity. The structure-function relationships governing antioxidant activity are remarkably precise [62]. Muñoz-Castiblanco et al. [63] demonstrated that *Lentinula edodes* crude polysaccharides (LECP) containing α/β glycosidic bonds showed EC_50_ values ranging from 0.51 to 3.59 mg·mL^−1^ across various radical scavenging assays, with the structural configuration directly influencing activity levels. The presence of (1→3;1→6) β-D-glucan linkages particularly enhances radical scavenging and reducing power capabilities [64].

β-glucans deserve special attention for their unique antioxidant mechanisms [65]. Yehia [66] reported that isolated β-glucan fractions from *L. edodes* at optimal doses of 3.5 mg·mL^−1^ produced 75.3% radical scavenging activity, 80.9% peroxyl radical inhibition, and 88% reduction of aflatoxin B1, demonstrating dose-dependent antioxidant and detoxifying effects. Unlike simple phenolic compounds that work primarily through direct radical scavenging, β-glucans can chelate metal ions that catalyze oxidative reactions, effectively preventing the initiation of lipid peroxidation cascades [67].

When compared to other natural antioxidants from plant, algal, or bacterial sources, mushroom-derived compounds often demonstrate superior stability and broader pH tolerance [68]. The molecular weight of these polysaccharides, typically ranging from 111.3 kDa to several hundred kDa, contributes to their stability and prolonged antioxidant activity [69]. Additionally, the synergistic interactions between different mushroom compounds can create antioxidant effects that exceed the sum of their individual contributions [70].

### 4.3. Antimicrobial, Antifungal, and Antiviral Effects

The antimicrobial arsenal within mushroom residue reflects nature’s sophisticated defense strategies, offering packaging developers bioactive compounds with diverse modes of action [71]. The primary mechanisms through which mushroom-derived compounds exert their antimicrobial effects include membrane disruption, enzyme inhibition, and reactive oxygen species (ROS) generation, each targeting different aspects of microbial physiology [72]. Chitosan, derived from mushroom chitin, demonstrates its antimicrobial activity primarily through electrostatic interactions with negatively charged bacterial cell walls [73]. Khubiev et al. [74] reported that chitosan-based films achieved significant antimicrobial efficacy, with the degree of deacetylation directly influencing antimicrobial potency. The mechanism involves disruption of membrane integrity, leading to leakage of intracellular contents and eventual cell death [75]. Bajrami et al. [76] demonstrated that chitosan and its methylated derivatives showed enhanced antimicrobial efficiency against *Lentilactobacillus parabuchneri* biofilms, with the antimicrobial spectrum extending beyond bacteria to include fungi and some viruses.

Phenolic compounds from mushroom residue operate through different mechanisms, often targeting specific enzymes essential for microbial metabolism [77]. Singh et al. [78] reported that phenolic acids in active packaging systems can inhibit key enzymes in bacterial respiratory chains, effectively starving microorganisms of energy. Shiitake (*L. edodes*) residue extracts showed minimum inhibitory concentrations (MICs) against *Pseudomonas aeruginosa*, *Escherichia coli*, and *Salmonella enterica* of 15, 7.5, and 7.5 mg·mL^−1^, respectively [79].

The synergistic effects among mushroom-derived compounds represent one of their most valuable characteristics for packaging applications [80]. Zhang et al. [81] demonstrated that ultrasonically functionalized chitosan-gallic acid films showed enhanced antimicrobial activity against *Staphylococcus aureus* through envelope disruption under UVA light exposure, with the combination effect exceeding individual component activities. Hao et al. [82] reported that antimicrobials immobilized on packaging films create coordinated antimicrobial mechanisms that reduce the likelihood of resistance development.

Real-world applications have demonstrated the practical value of these antimicrobial properties [83]. Packaging films incorporating mushroom extracts have successfully extended the shelf life of fresh produce, dairy products, and meat products by 30–50% compared to conventional packaging, showcasing not only efficacy but also compatibility with food contact applications and consumer safety requirements [84].

### 4.4. Barrier and UV-Protective Properties

The barrier properties of mushroom-derived compounds stem from their natural role as protective barriers in fungal cell walls and fruiting bodies [84]. Phenolic compounds, with their conjugated aromatic systems, provide excellent UV absorption properties, effectively filtering harmful wavelengths that can degrade food nutrients and packaging materials [85].

Gennaro et al. [86] demonstrated that UV-shielding biopolymer coatings loaded with bioactive compounds from natural sources significantly improved UV protection in food packaging applications. The extended conjugation in compounds like ferulic acid and vanillic acid allows them to absorb UV radiation across both UVA and UVB ranges, providing comprehensive photoprotection [87].

The recovery of industrial mushroom-based residues is part of the zero-residue circular economy. Lentil seed coats are generally considered residue. However, this low-value residue is rich in bioactive compounds and may be considered an eco-friendly source of health-promoting phytochemicals. For the first time, a sustainable microwave-assisted extraction technique was applied, and a solvent screening was conducted to enhance the bioactive compound content and antioxidant activity of green and red lentil hull extracts [88].

Furthermore, chitosan films impregnated with natural extracts showed enhanced UV-shielding properties, with UV transmittance reduced by up to 90% compared to control films, while maintaining transparency in the visible light range. Chitosan contributes to barrier properties by forming dense, well-ordered films with low free volume [87]. Hydrogen bonding between chitosan chains forms a tight network that restricts the movement of water vapor and gases [89]. Santos and Martins [90] demonstrated that multifunctional films blended with polyphenol-rich extracts showed improved barrier properties, with water vapor permeability reduced by 40–60% compared to neat polymer films.

The effects on water vapor permeability are significant for food packaging applications [91]. Dang et al. [92] reported that chitosan/polyvinyl alcohol films enhanced with natural extracts demonstrated selective permeability, allowing controlled moisture transfer that prevents both dehydration and excessive moisture accumulation. This selective barrier behavior is crucial for maintaining optimal humidity levels around fresh produce and preventing condensation that could promote microbial growth [93]. Light stability represents another critical barrier property enhanced by mushroom compounds [94].

The natural pigments and phenolic compounds in mushroom extracts can absorb and dissipate light energy harmlessly, protecting both the packaging material itself and the enclosed food products from photodegradation [95]. This photoprotective effect can significantly extend product shelf life, particularly for light-sensitive nutrients like vitamins and essential fatty acids [96].

### 4.5. Multifunctional and Synergistic Effects

The true power of mushroom-derived packaging materials emerges when their various functional properties work in concert, creating multifunctional systems that address multiple preservation challenges simultaneously [97]. This multifunctionality is not merely additive but often synergistic, with different bioactive compounds enhancing each other’s effectiveness through complementary mechanisms [98]. The combination of antioxidant, antimicrobial, and barrier properties creates a comprehensive preservation system that targets the primary causes of food deterioration [99]. Ordoñez et al. [100] demonstrated that biodegradable active materials containing phenolic acids for food packaging applications showed enhanced performance when multiple bioactive compounds were present, with preservation effects exceeding what any single approach could achieve. Extraction and modification processes play crucial roles in determining the multifunctional profile of mushroom-derived materials [101]. Mild extraction conditions may preserve the native structure and activity of bioactive compounds, while more intensive processing can create new functional properties through chemical modifications [102]. Controlled processing techniques are essential for achieving desired functional balances without compromising bioactivity [103].

One of the most significant challenges in developing multifunctional mushroom-based packaging is balancing mechanical strength and bioactivity retention [104]. High concentrations of bioactive compounds may compromise the mechanical properties of packaging films, while processing conditions that optimize mechanical strength might degrade sensitive bioactive molecules [105]. Successful formulations require careful balance, often achieved through innovative processing techniques like microencapsulation or layer-by-layer assembly [106].

### 4.6. Structure–Function Insights

Understanding the relationship between molecular structure and functional performance is essential for optimizing mushroom-derived packaging materials [107].

The correlation between molecular composition and functional performance follows predictable patterns that can guide material design and processing decisions [108]. Flores et al. [109] reported that the degree of branching in β-glucan polysaccharides directly influences their film-forming properties and mechanical strength, while hydroxyl content determines hydrophilicity and barrier characteristics. Polymer compatibility emerges as a critical factor determining the ultimate performance of mushroom-derived packaging materials [110]. The molecular weight, polarity, and functional group distribution of mushroom compounds must complement those of the base polymer matrix to achieve uniform dispersion and optimal property development [111]. Incompatible systems often exhibit phase separation, leading to heterogeneous properties and reduced performance [112]. Particle size and dispersion quality significantly impact bioactivity retention and mechanical properties [113]. Nanoscale dispersion of mushroom-derived compounds can enhance their surface area and bioavailability, potentially improving functional performance [114]. However, achieving and maintaining nanoscale dispersion requires careful control of processing conditions and may require the use of dispersing agents or surface modifications [115].

The integration of these functional properties into practical packaging systems requires careful consideration of processing conditions, storage stability, and end-use requirements [116]. The multifunctional nature of mushroom-derived compounds offers unique opportunities to develop packaging materials that address multiple preservation challenges while supporting sustainability goals through residue valorization and biodegradability [117].

**Figure 4:**
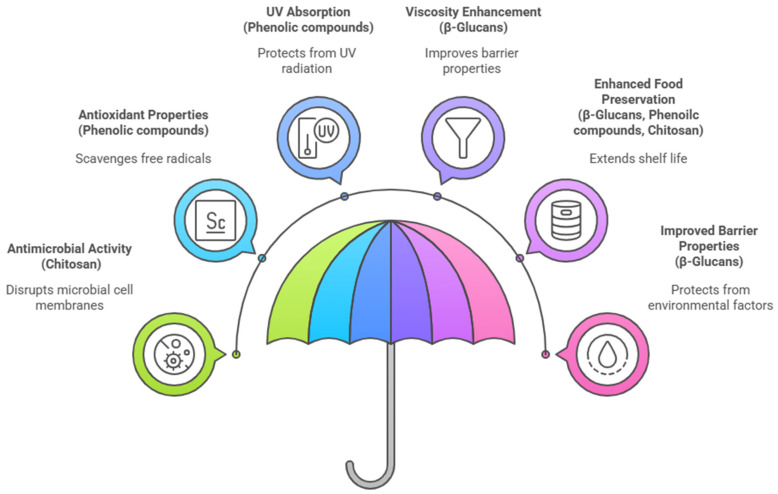
Bioactive compounds derived from mushroom residues and their functional property and packaging performance.

Functional properties of mushroom extracts in packaging films can be summarized in Table 2, whereas Figure 4 summarizes the bioactive compounds derived from mushroom residues and their functional property and packaging performance.

## 5. Extraction, Fractionation, and Modification Techniques

### 5.1. Green Extraction Methods

The recovery of bioactive compounds from mushrooms residues through green extraction strategies stands out because plant-derived substances retain their stability after extraction, allowing for longer sustainability in food packaging [18]. These approaches help preserve the structural stability and functional properties of plant- and fungus-derived substances, thereby supporting longer-term sustainability in food packaging applications [118]. Compared to conventional extraction, green methods significantly reduce extraction time and energy consumption [119]. Extraction techniques such as ultrasound-assisted extraction (UAE), microwave-assisted extraction (MAE), pressurised liquid extraction, supercritical fluid extraction, enzyme-assisted extraction, and pulsed electric field extraction have been employed for the recovery of antioxidants from plant residues [19]. The most extensively used procedures in the emerging industry for solid substrates are MAE and UAE. Their key operational features, advantages, and applications are summarized in Table 3.

### 5.2. Process Optimization and Scalability

Scaling up the use of mushroom-derived bioactive compounds in sustainable packaging requires more than just efficient extraction; it demands careful refinement at every stage. After extraction, fractionation, and stabilization steps, which help isolate valuable compounds such as polysaccharides, phenolics, and proteins while protecting their functional integrity [124,125]. Techniques such as membrane separation and chromatography are especially effective for this purpose [126].

To make these bioactives compatible with packaging polymers, chemical and physical modifications, such as blending, grafting, and crosslinking, are commonly employed [118]. These adjustments improve material strength, water resistance, and shelf-life performance [122]. However, moving from lab to industry is not straightforward. Challenges around throughput, energy costs, and reproducibility make process optimization essential [119]. Emerging solutions, such as modular setups and semi-continuous systems, offer more flexibility for scale-up [88].

**Figure 5:**
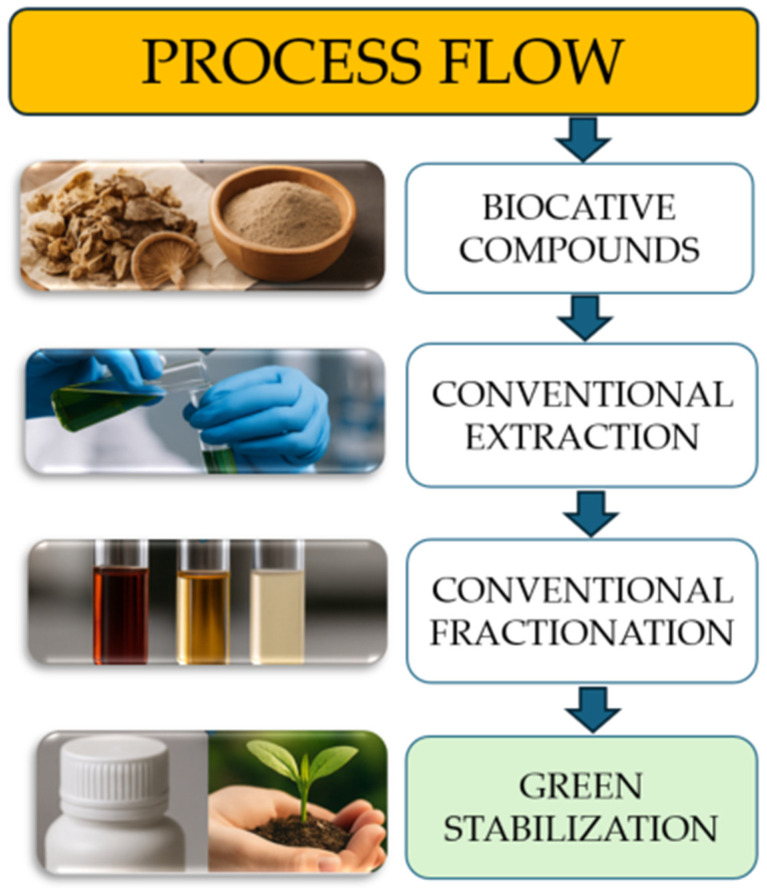
Process flow: conventional vs. green extraction techniques.

Green extraction technologies, particularly UAE and MAE, stand out for their speed, low energy consumption, and retention of compound bioactivity [127,128]. These approaches significantly contribute to the development of efficient and environmentally friendly bio-packaging systems. The process, illustrated in Figure 5, begins with the extraction of bioactive compounds from residual mushroom biomass, which is rich in antioxidants, polysaccharides, and phenolic compounds [129]. As depicted, the workflow proceeds through several key stages: conventional extraction, fractionation, and green stabilization to obtain functional materials suitable for sustainable packaging applications.

These compounds are then subjected to conventional extraction and fractionation steps, during which they are isolated and separated based on their unique properties [130]. As shown in the diagram, stabilization is achieved through a two-step process: first, traditional methods are employed, followed by a transition to green stabilization, highlighted in green to emphasize its eco-friendly nature. This final step ensures the compounds remain effective while minimizing environmental impact [131]. Altogether, the process not only gives new life to agricultural residue but also leads to biodegradable packaging solutions that are safer for both consumers and the planet.

## 6. Integration into Biopolymers and Packaging Films/Coatings

### 6.1. Incorporation Strategies (Blending, Coating, Grafting)

The methods for incorporating biopolymers into packaging films and coatings play a crucial role in determining their performance characteristics, as summarized in Table 4. Each strategy —blending, coating, grafting, and in situ polymerization—offers distinct advantages in terms of distribution, interaction, and structural modification.

These modifications are primarily determined by the quantity and nature of the biopolymer, allowing adjustments to mechanical, thermal, barrier, and optical properties [136].

### 6.2. Effects on Packaging Properties

The incorporation of biopolymers, such as essential oils, polysaccharides, and proteins, via these strategies results in significant changes in the physical and functional properties of packaging films. Mechanical properties, such as tensile strength, can either decrease or increase depending on the biopolymer and method used. For instance, polyvinyl alcohol films blended with chitosan, gelatin, and citrate exhibit a reduction in tensile strength of approximately 15%, whereas grafting polycaprolactone onto gregory bark has been shown to enhance tensile strength by 127% [137].

Changes in optical properties also vary with the incorporation technique. Blending tends to result in minimal alterations, often maintaining transparency, whereas surface deposition or chemical grafting can render the films more opaque due to scattering effects from the added materials [138].

### 6.3. Bioactivity Retention and Safety Aspects

A critical advantage of incorporating biopolymers is the retention of their bioactive properties, which can contribute to extended shelf life and enhanced safety of packaged goods. Biopolymers can facilitate sustained release of active compounds when stably incorporated into the polymer matrix, thereby preserving their functional efficacy over time [139].

However, the introduction of biopolymers also raises safety concerns, particularly regarding their biodegradability and interactions with food products. Unlike inert synthetic polymers, biopolymers may degrade during storage, potentially releasing harmful byproducts. Additionally, migration of additives, especially during sterilization or contact with liquids, poses risks that are closely monitored by regulatory bodies such as the European Food Safety Authority (EFSA) and the U.S. Food and Drug Administration (FDA) [140]. Packaging materials must thus ensure structural integrity, prevent microbial contamination, and resist degradation throughout the product’s shelf life [141]. Formulations typically include polyethylene or polypropylene as the matrix, along with plasticizers, colorants, and active agents [142]. While functional additives enhance bioactivity, their stability and safety must be rigorously assessed to meet regulatory standards and consumer expectations for safety [143].

## 7. Case Studies and Emerging Applications

Among fungal-based packaging technologies, mycelium composites are the most commercially advanced. Ecovative’s molded foams, developed from mycelium and agricultural residues, have been validated at an industrial scale, including use by Dell and IKEA, making them strong candidates to replace expanded polystyrene [144]. In contrast, active and edible films, such as oyster mushroom–based antioxidant coatings [145] and shiitake pullulan oxygen-barrier films [146], remain largely experimental. Their functional promise is clear, but issues of stability, storage, and scalability continue to prevent market readiness. Mushroom leather analogs, pioneered by MycoWorks and Bolt Threads, demonstrate flexibility and durability comparable to that of animal leather; however, their current impact is concentrated in fashion and luxury applications rather than the food system. High production costs remain a barrier to wider use [147]. Hybrid systems, such as chitosan–starch blends, offer improved tensile strength and barrier performance compared to single-component fungal films. However, they complicate regulatory approval processes since multi-polymer formulations require additional validation [148]. These examples are summarized in Table 5, which outlines key case studies, their respective applications, features, and potential impact across the fungal packaging landscape.

Overall, the field shows uneven maturity. While some technologies (e.g., mycelium foams) are already operating at a commercial scale [18], others (e.g., edible and active films) remain at the pilot or laboratory stage [146,147]. This divergence underscores that functionality alone does not guarantee adoption; cost-effectiveness, regulatory compliance, and consumer perception are equally decisive factors. The contrast highlights the uneven maturity of fungal packaging technologies, where market success depends not only on bioactivity but also on cost-efficiency, regulatory acceptance, and consumer perception.

Additionally, new directions are emerging at the intersection of fungal biotechnology and circular economy models [149]. Recent studies have investigated the valorization of spent mushroom substrate (SMS) as a feedstock for biodegradable films, potentially lowering raw material costs and diverting residue streams from landfills [11]. Similarly, fungal-derived nanocellulose and β-glucan composites are being explored for their advanced barrier and mechanical properties, indicating potential applications in next-generation smart packaging [150]. Pilot projects integrating fungal coatings with conventional biodegradable polymers (e.g., PLA) also show potential to enhance moisture resistance without compromising compostability [151].

Despite these innovations, scaling remains a bottleneck. Start-ups and research groups demonstrate technical feasibility, yet few have bridged the gap to mass production due to high manufacturing costs, processing variability, and limited regulatory precedents [151,152]. The translation of fungal-based packaging from niche applications (e.g., electronics, luxury fashion) to mainstream food systems will likely depend on further cost optimization, harmonized safety standards, and strategies to build consumer trust in bio-based alternatives [153].

## 8. Challenges, Trade-Offs, and Knowledge Gaps

**Figure 6:**
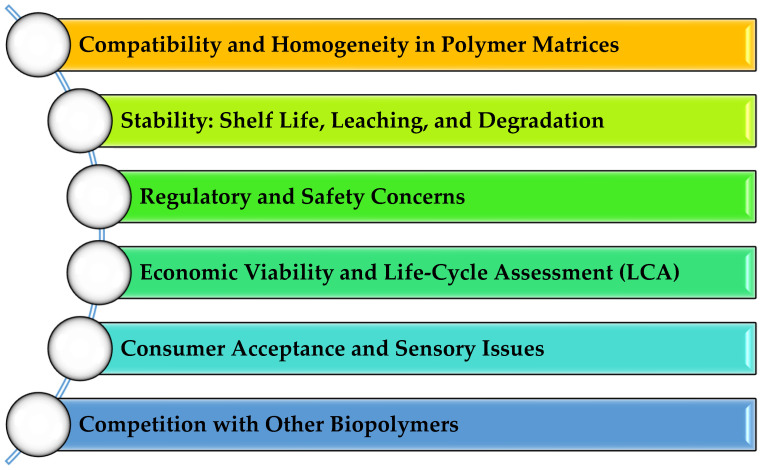
Critical Barriers to the Application of Mushroom-Derived Biopolymers.

Although mushroom residues hold significant promise for sustainable packaging, several challenges must be resolved before large-scale adoption and commercial viability can be achieved. These barriers encompass issues of compatibility, stability, regulatory compliance, economic feasibility, and consumer perception, all of which shape the integration of mushroom-derived bioactives into food packaging systems. A visual overview of these critical factors is provided in Figure 6.

### 8.1. Technical and Stability Challenges

Compatibility and dispersion remain key hurdles when incorporating mushroom-derived compounds such as polysaccharides, proteins, and phenolics into existing polymer matrices. Achieving homogeneity is vital to ensure consistent mechanical and barrier properties [154]. Hernando et al. (2023) emphasize the importance of optimized blending and processing methods to enhance integration, while variations in solubility and polarity necessitate customized compatibilization strategies to achieve stable formulations [155].

Another critical limitation is stability. Bioactive compounds are prone to leaching and degradation during storage, resulting in a shortened shelf life and compromising their functional reliability [156,157]. As noted by Silva et al. (2020), maintaining the stability of phenolics and β-glucans throughout film formation and storage is fundamental to long-term effectiveness. Strategies such as encapsulation, cross-linking, and controlled release are currently under investigation to address these weaknesses [158].

Furthermore, scalability of these stability solutions is a challenge in itself [34,159,160,161]. High-energy processing methods may degrade thermally sensitive compounds, whereas low-energy alternatives may fail to provide sufficient polymer dispersion [162]. Addressing these trade-offs requires advanced engineering approaches, such as integrating nanocomposites or blending with other natural polymers, to balance functionality with stability [163].

While laboratory-scale formulations demonstrate promising results, transferring these strategies to industrial processes often results in inconsistent film morphology and reduced reproducibility. For instance, Optical and mechanical properties are usually sensitive to microstructural nonuniformities. The same irregularities that originate from dispersion instability or drying artifacts can produce local stress concentrations or weak points. In “Morphological Characteristics of Biopolymer Thin Films,” Băbuțan et al. (2024) emphasize that defects or uneven film thickness degrade barrier and mechanical metrics [164].

### 8.2. Regulatory and Safety Barriers

From a regulatory perspective, the introduction of novel bio-based materials into food-contact applications must comply with strict safety standards. Despite promising bioactivities, mushroom-derived extracts may require extensive toxicological validation [165]. Grimm’s standards further complicate the commercialization process [166]. While some jurisdictions provide clear frameworks for evaluating natural biopolymers, others remain ambiguous, leading to delays in approval processes [167]. This regulatory uncertainty may deter investment and slow innovation [168]. Moreover, the heterogeneous composition of mushroom residues makes it challenging to establish uniform safety benchmarks, as variability in fungal species, cultivation methods, and extraction protocols can yield different toxicological profiles [150].

Emerging research also highlights the importance of life-cycle safety assessments [42]. Beyond initial food-contact toxicity evaluations, long-term studies on biodegradation, environmental release of metabolites, and interactions with other food packaging components remain limited [169]. Without comprehensive data, regulators may adopt a precautionary stance, imposing stricter compliance barriers that hinder the adoption of materials derived from mushrooms [170].

### 8.3. Economic Feasibility and Consumer Acceptance

Economic aspects remain a critical factor in the development and implementation of mushroom-derived materials. Although mushroom residues are low-cost feedstocks, the associated processes of extraction, purification, and formulation often entail significant expenses [2]. Sevigné-Itoiz et al. (2021) emphasize that robust life-cycle assessments (LCAs) are crucial for capturing both the environmental and economic implications of such materials [171]. In contrast, Grimm and Wösten (2018) argue that incorporating these residues into a circular economy model can enhance overall cost efficiency. By valorizing residue and reducing reliance on virgin raw materials, this approach could offset processing costs and contribute to sustainable resource management [172].

Beyond the technical and financial challenges, consumer perception also significantly influences market viability. Mushroom-based films may exhibit atypical sensory attributes, such as unusual colors, textures, or odors—that could discourage consumer adoption [173,174,175]. Kirtil et al. (2025) note similar concerns in the context of alternative proteins, where off-flavors, astringency, and undesirable aromas hinder acceptance. They advocate for advanced molecular and biochemical processing strategies to mitigate these sensory drawbacks [176].

At the same time, Cottet et al. (2020) observe that the field of microbial biomass-based bioplastics is rapidly evolving. They suggest that for mushroom-derived materials to remain competitive, they must carve out a unique position by emphasizing their multifunctional properties and ecological benefits [177].

**Figure 7:**
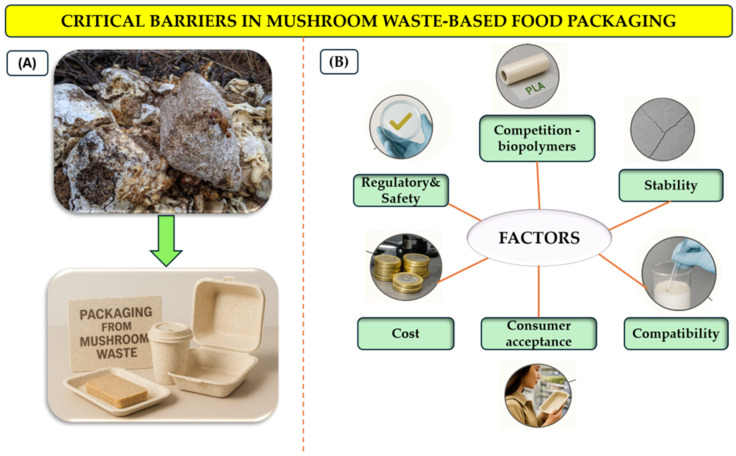
Critical Barriers to the Application of Mushroom-Derived Biopolymers in Food Packaging: (**A**) Before (starter products-spent mushroom substrate (SMS) and after manufacturing packaging materials (final products); (**B**) Factors affecting the packaging manufacturing process.

In summary, while mushroom residues hold substantial promise for eco-friendly packaging solutions, their practical application hinges on overcoming a series of complex barriers, ranging from technical feasibility and regulatory compliance to consumer perception and economic scalability. Interdisciplinary research and targeted innovation will be pivotal in bridging these gaps. If successfully addressed, these efforts could establish mushroom-based materials as a cornerstone of the emerging circular, bio-based packaging paradigm (Figure 7).

Advancements in biofabrication and synthetic biology enable the tailoring of mushroom strains for high-yield production of compounds [153]. Controlled cultivation can enhance the properties of derived materials [178]. Smart packaging technologies integrating sensors or indicators with biodegradable mushroom polymers allow real-time monitoring of food quality [179]. Artificial intelligence can optimize extraction and logistics in circular systems [180]. Digital platforms improve coordination between residue generators and users [181].

**Figure 8:**
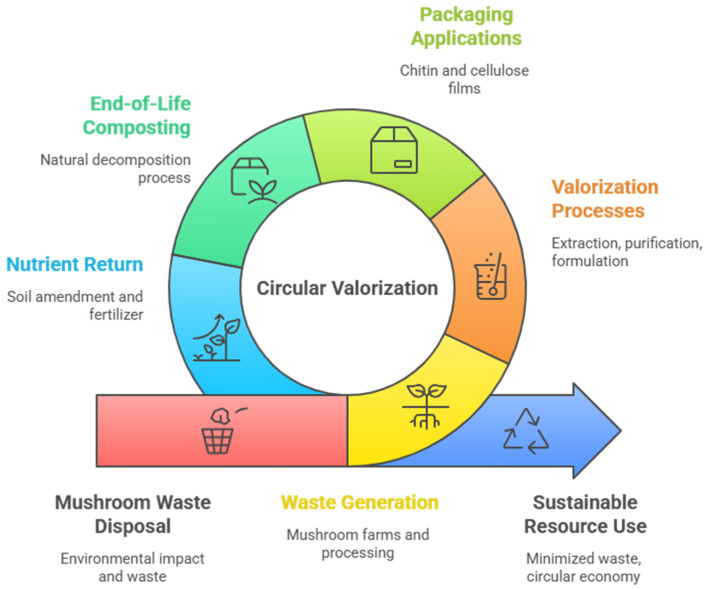
Circular economy pathway for mushroom residue valorization: from residue generation to sustainable food packaging and nutrient recovery.

More challenges and opportunities in mushroom residue valorization can be listed in Table 6, whereas Figure 8 summarizes circular economy pathway for mushroom residue valorization: from residue generation to sustainable food packaging and nutrient recovery.

The valorization of mushroom residue for packaging applications embodies a powerful intersection of environmental urgency and economic opportunity [182]. This review has demonstrated that mushroom-derived packaging materials offer a viable solution to multiple sustainability challenges by uniting functional performance, ecological impact reduction, and resource efficiency [183].

To fully realize this potential, strategic priorities must align across three core domains:

  (i). technological innovation to enhance functionality and cost-effectiveness;
 (ii). infrastructure development to enable scalable, efficient processing; and
(iii). regulatory support to foster market readiness and consumer acceptance [184].

A successful transition toward mushroom-based packaging requires collaborative engagement across the value chain—from cultivation and residue management to material processing and final packaging deployment [185]. Building reliable supply chains, ensuring consistent product quality, and delivering measurable environmental and economic benefits will be critical to stakeholder adoption [186]. The insights presented throughout this section underscore the transformative potential of mushroom residue valorization as a cornerstone of sustainable packaging innovation [187].

## 9. Sustainability, Circular Economy, and Valorization Pathways

### 9.1. Positioning Mushroom Residue in the Circular Bioeconomy

Positioning Mushroom Residue in the Circular Bioeconomy The mushroom industry exemplifies circular bioeconomy principles by transforming residue into valuable inputs for further production cycles [188]. In recent years, valorizing residue and cascading resource use has gained importance as a way to address environmental issues and generate economic value from agricultural residues [189]. Mushroom farming generates large amounts of spent mushroom substrate (SMS) and non-marketable fruiting bodies, typically 5–7 kg of residue per kg of fresh mushrooms [190].

This shift from a linear “take-make-dispose” model to regenerative systems represents a key part of the circular economy [190]. Initiatives such as the FUNGUSCHAIN EU project have shown how mushroom offcuts can be systematically valorized to extract various useful components [191]. This approach aligns with circular economy principles by extending material use, maximizing value, and enabling recovery and regeneration at end-of-life [192]

Mushroom-derived materials are particularly well-suited for biodegradable packaging due to their natural degradability and environmental compatibility [193]. Unlike petroleum-based packaging, mushroom-based materials can break down in controlled settings and return nutrients to the soil, closing the material loop [194]. As such, valorizing mushroom residue supports sustainable packaging solutions that tackle both environmental and residue management goals [195].

### 9.2. Cascading Valorization Strategies

Mushroom residue can be used to sequentially recover bioactives, biopolymers, and composites, which maximizes its utility [196]. For example, non-marketable edible mushrooms have been used in biorefineries to produce chitin (2.1% yield), citric acid (15.2%), and ethanol (0.31 g/g glucose), demonstrating economic viability alongside environmental benefits [197]. Pilot-scale applications have validated these multi-product systems [198]. One such model integrated mushroom cultivation with ethanol production and co-product recovery, such as animal feed biomass and lignin-rich soil enhancers [199].

Another commercial-scale model proposes a zero-residue biorefinery that includes cellulase production, bioethanol generation, and bio-fertilizer creation [200]. These systems can extract various compounds from mushroom residue before final conversion to compost or biogas [201].

A cascading use strategy has shown that SMS can yield biogas (0.25 m^3^/kg volatile solids), vermicompost (68% efficiency), and biochar (32% yield), with total returns surpassing traditional disposal methods by 340% [202].

### 9.3. Life Cycle Assessment (LCA) and Environmental Footprint

LCA studies comparing mushroom-based and petroleum-based packaging reveal significant advantages for the former [203]. For instance, *Agaricus bisporus* production emits 2.55 × 10^4^ kg CO_2_-equivalent per 23,000 kg substrate [203]. Optimization could cut emissions by 5%, mainly through better energy use and substrate efficiency [204]. For shiitake mushrooms, GHG emissions are 2.38 kg CO_2_-equivalent per kg of product, with energy consumption being the primary contributor (72.87%) [205]. Beyond carbon emissions, mushroom residue as fertilizer significantly reduces environmental impacts across various categories, including land use (45%), fossil fuel use (38%), ecotoxicity (52%), and climate change effects (28%) compared to synthetic fertilizers [205].

### 9.4. Industrial Symbiosis and Cross-Sector Integration

Integrating mushroom residue with other agricultural residues can support regional biorefineries [206]. One example is composting mushroom residue with other materials to create bioenergy pellets (18.2 MJ/kg energy, <10% moisture) [207]. This enables regional processing hubs for mixed residue streams and standardized bio-products.

Field studies in Vietnam revealed 20 active valorization pathways in mushroom production, from direct land application to complex bioactive extraction [208]. These diverse strategies highlight the adaptability of mushroom residue utilization.

Scaling up such systems requires infrastructure and policy support, such as collection and transport systems, processing facilities, and quality control [209]. Coordinated policy, recognition of bio-based materials, financial incentives, and industry standards are all essential. Programs like the EU Circular Economy Action Plan are paving the way for this transition [210].

### 9.5. Economic and Social Dimensions

Economic analyses consistently show strong returns from mushroom residue valorization when multiple outputs are considered [211]. For example, integrated biorefineries can achieve net present values of $2.85 million over 10 years, with a payback period of 4.2 years and IRR > 25% [197].

Scalability depends on achieving economies of scale while maintaining quality and demand [212]. Facilities serving 500–1000 farms are considered optimal, with transportation accounting for 15–20% of costs [213]. Centralized systems offer efficiency, while decentralized models provide flexibility.

Social benefits include job creation in processing, logistics, and marketing. These systems support rural economies and local agriculture [214]. Surveys indicate high consumer interest in sustainable packaging, with 68% willing to pay more for bio-based options [173]. However, market success still depends on packaging performance, durability, and cost competitiveness [215].

## 10. Conclusions

Harnessing mushroom residues offers a promising route to creating sustainable, functional food packaging. These residues, rich in bioactive compounds such as β-glucans, phenolics, proteins, and chitin, offer a range of beneficial properties, improving the strength, barrier function, and bioactivity of biodegradable films and coatings. Through green extraction techniques, these often-overlooked materials can be efficiently repurposed into high-value ingredients, supporting both environmental goals and circular economy principles. However, key challenges must be overcome before such packaging becomes mainstream. Stability of the compounds, compatibility with polymers, scalability of the extraction process, regulatory hurdles, and consumer perception remain critical obstacles. Progress in these areas will require interdisciplinary collaboration, linking food science, materials engineering, and life-cycle assessment, alongside comparative evaluations with other agricultural residues.

Ultimately, turning mushroom residue into functional packaging is both a sustainable imperative and a technological opportunity. It enables the mushroom industry to reduce plastic use, generate new income streams, and support the transition toward a circular bioeconomy.

## Figures and Tables

**Table 1:** Physicochemical and microbiological characteristics of spent mushroom substrate and their Relevance to valorization.

Characteristic	Typical Values (SMS Basis)	Relevance to Valorization
Moisture content	60–70% (wet basis)	High water-holding capacity is ideal for composting or soil amendment; it requires drying for bioenergy use.
Organic matter	40–60% dry weight (DW)	Supports biodegradation and biogas production (e.g., yields 200–300 mL/g volatile solids)
pH	6.5–8.0	Neutral to slightly alkaline conditions facilitate nutrient availability in soil amendments and microbial fermentations
Nitrogen (N), phosphorus (P), potassium (K)	N: ~1–2%; P: 0.3–1.0%; K: 0.5–2.0% (DW)	Nutrient-rich for fertilizer use; can improve crop performance
Lignocellulose (cellulose, hemicellulose, lignin)	Lignin fraction often > 15%	Lignin and cellulose fractions can be used for bioenergy, biochar, or material production.
Microbial load	10^6^–10^8^ CFU/g	Contains diverse decomposers (bacteria, fungi) useful in composting, bio-augmentation, and enzyme recovery

Sources: [22,24].

**Table 2:** Functional Properties of Mushroom Extracts in Packaging Films.

Compound Class	Primary Function	Quantitative Performance	Key Mechanisms	Refs.
Chitosan	Antimicrobial, Barrier	90–99% microbial reduction; 40–60% barrier improvement	Membrane disruption, Film formation	[73,74,75,76]
β-glucans	Antioxidant, Film-forming	IC50: 0.52–3.59 mg·mL^−1^; 75–88% radical scavenging	Radical scavenging, Metal chelation	[63,64,66]
Phenolic compounds	Antioxidant, UV protection	MIC: 7.5–15 mg·mL^−1^; 90% UV reduction	Electron donation, UV absorption	[78,79,86]
Polysaccharide fractions	Multifunctional	82–94% antioxidant activity; Enhanced tensile properties	Multiple radical scavenging mechanisms	[94]

**Table 3:** Comparison of Ultrasound-Assisted and Microwave-Assisted Extraction Techniques.

Aspect	Ultrasound-Assisted Extraction (UAE)	Microwave-Assisted Extraction (MAE)
Driving Principle	Acoustic cavitation (formation and collapse of microbubbles)	Dielectric heating (direct microwave energy absorption by solvents and samples)
Mechanism of Action	Cell disruption via fragmentation, pore formation, and sonoporation	Rapid heating of intracellular moisture, disrupting compound–matrix bonds
Effect on Plant Cell Walls	Damages the polysaccharide network; enhances the release of intracellular compounds.	Heat enhances the solubility and diffusion of compounds.
Efficiency	High mass transfer and solvent penetration; fast release	Quick and uniform heating; high extraction efficiency
Temperature Control	Generally moderate; no need for high temperatures	Can exceed the solvent boiling point in closed vessels without decomposition
Applications	Fruit, vegetable, and mushroom residues for bioactive compound recovery	Broad use for thermally stable bioactives; efficient for high-value extracts
Solvent Interaction	Mechanical effects enhance solvent penetration	Solvents absorb microwave energy for direct heating
Advantages	Eco-friendly, energy-efficient, effective at room temp, enhances yield	High-speed, energy-efficient, enhanced extraction at controlled high temps
References	[120,121]	[122,123]

**Table 4:** Overview of Biopolymer Incorporation Methods into Packaging Films and Coatings.

Method	Description	Advantages	Ref.
Blending	Physical mixing of a bio-based additive with the polymer matrix. The additive is mixed into the melt or solvent-blended before the film is formed.	Uniform distribution of the additive retains the base polymer structure.	[132]
Coating	Application of a biopolymer layer onto the surface of an existing film. Spraying or spreading a layer on the film surface.	Modifies only the surface layer; does not affect the film core.	[133]
Grafting	Chemical modification of polymer chains to enable bonding with the film surface. Covalent bonding through chain-end or side-chain functionalization.	Stable and durable attachment; improves interfacial properties.	[134]
In situ polymerization	Polymerization of bio-based molecules directly within the packaging matrix. Monomers are dispersed in the medium and polymerize in place during matrix formation.	Ensures homogeneous integration; strong interaction between the biopolymer and the matrix.	[135]

**Table 5:** Innovative Applications in the Field of Residue Valorization.

Application Area	Case Study/Example	Key Features	Impact/Potential	Ref.
Mycelium composites	Ecovative Design packaging for electronics	Molded foam-like structures from mycelium + agri-residue	Biodegradable replacement for EPS foams	[144]
Mushroom leather analogs	MycoWorks, Bolt Threads	Flexible, leather-like fungal mats	Premium, sustainable alternative to leather/plastics	[147]
Active packaging	Oyster mushroom-based films	Antioxidant + antimicrobial activity	Shelf-life extension for fruits & vegetables	[145]
Edible coatings	Shiitake pullulan films	Transparent, oxygen-barrier edible films	Reduced food residue, no secondary packaging needed	[146]
Hybrid biopolymer systems	Chitosan-starch blends	Stronger tensile and barrier properties	Expanded functional range of biofilms	[148]

**Table 6:** Challenges and opportunities in mushroom residue valorization.

Challenge Category	Specific Issues	Potential Solutions	Economic Impacts
Technical	Processing efficiency, Product standardization	Advanced extraction technologies, Quality control systems	15–25% cost reduction
Economic	Scale-up costs, Market development	Regional processing hubs, Policy incentives	$2.85 M NPV over 10 years
Environmental	Energy consumption, Water usage	Renewable energy integration, Process optimization	28% GHG reduction
Social	Consumer acceptance, Skill development	Education programs, Training initiatives	68% consumer willingness to pay premium
Regulatory	Standards development, Certification	Harmonized regulations, Industry standards	Reduced compliance costs

## Data Availability

No new data were created or analyzed in this study.
